# Assessment of minimum discard volume for central venous catheter sampling: Validation via busulfan therapeutic drug monitoring in pediatric transplant patients

**DOI:** 10.3389/fped.2026.1925821

**Published:** 2026-09-14

**Authors:** Fang Ye, Huiying Ye, Dongwei Cui, Xuanxuan Zhang, Sailan Luo, Yuan Luo, Sandip Patil

**Affiliations:** 1 Department of Hematology and Oncology, Shenzhen Children's Hospital, Shenzhen, Guangdong, China; 2 Department of Pharmacy, Shenzhen Children's Hospital, Shenzhen, Guangdong, China

**Keywords:** blood conservation, blood sampling, Busulfan, central venous catheters, pediatric hematopoietic stem cell transplantation, therapeutic drug monitoring

## Abstract

**Purpose:**

Frequent blood sampling in pediatric patients, particularly during hematopoietic stem cell transplantation (HSCT), can lead to significant cumulative blood loss and increase the risk of iatrogenic anemia. This study aimed to evaluate whether reducing the discard volume prior to blood sampling affects the accuracy of plasma Busulfan concentration measurements in this vulnerable population.

**Patients and methods:**

This prospective cohort study enrolled thirteen pediatric patients receiving HSCT at a tertiary children's hospital in China between July and October 2024. Blood samples were collected via double-lumen central venous catheters at seven time points (T0–T6) after the fourth dose of Busulfan, using four different discard volumes (1, 2, 3, and 4 mL). Each patient contributed repeated measurements under all four discard-volume conditions, resulting in 364 samples. Paired analyses were used to account for the within-patient repeated-measures design, and AUC non-inferiority was assessed against the prespecified 6% margin using the lower bound of the two-sided 95% confidence interval for the paired differences.

**Results:**

Using paired within-patient analyses, no significant differences in plasma Busulfan concentrations were observed among the discard-volume conditions. Paired differences between reduced discard volumes (1, 2, and 3 mL) and the 4 mL reference remained small, with 95% confidence intervals demonstrating consistent agreement. AUC non-inferiority analysis showed that the reduced discard-volume approaches remained within the prespecified 6% non-inferiority margin. ICC demonstrated excellent agreement between the reduced discard-volume conditions and the 4 mL reference for both individual time-point concentrations (ICC > 0.95) and AUC values. Bland–Altman analysis showed consistent agreement, with differences evenly distributed within the calculated 95% limits of agreement.

**Conclusion:**

A minimal discard volume of 1 mL demonstrated non-inferior accuracy compared with the standard 4 mL discard volume for determining plasma Busulfan levels and overall drug exposure during therapeutic drug monitoring. Implementing this approach may reduce cumulative discarded blood volume by up to 21 mL per patient during the seven sampling time points; however, the direct effect of reduced discard volume on anemia or transfusion requirements was not evaluated. This study adhered to the STROBE guidelines for observational research, and no patient or public contribution was involved.

## 1 Introduction

Hematopoietic stem cell transplantation (HSCT) represents a vital therapeutic approach for treating various hematological disorders, genetic disorders, and immune-deficiency illnesses (1). Within HSCT conditioning regimens, Busulfan, a bifunctional DNA alkylating agent, serves as a standard treatment in combination with cyclophosphamide or fludarabine (2). Given its narrow therapeutic window, Busulfan's systemic exposure is closely correlated with both therapeutic efficacy and toxicity (3). Consequently, therapeutic drug monitoring (TDM) is essential to ensure the safety and efficacy of treatment, particularly in pediatric populations. FDA-approved Busulfan dosing recommendations incorporate therapeutic drug monitoring and AUC-guided dose adjustment, and clinical transplantation centers generally follow this plan to implement individualized administration of Busulfan (4, 5). In clinical practice, patients undergoing HSCT commonly have a central venous catheter (CVC) placed for medication infusion and blood sampling. Compared to peripheral venipuncture, blood sampling via CVC reduces patient discomfort and improves the blood sampling success rates (6). However, blood sampling through CVC may be diluted by residual medications or 0.9% sodium chloride solution within the catheter-lumen, potentially compromising the accuracy of test results (7). Currently, the discard method is one of the most widely practiced techniques for blood sampling through CVC, in which a predetermined volume of blood is initially withdrawn and discarded before the actual blood sampling. This approach ensures that the obtained blood samples are not affected by catheter-related contamination or dilution (8). However, there is no consensus regarding the optimal discard volume. The latest Infusion Nursing Society (INS) guidelines recommend discard volumes ranging from 2 to 25 mL (7), while other studies report discard volumes of 3–5 mL for pediatric patients (9). For Busulfan TDM, this issue is particularly significant, as patients require blood sampling at seven time points (0, 1, 2, 2.5, 3, 4, and 6 h) following the fourth Busulfan infusion (10). Based on the commonly used discard volume of 3–5 mL, the cumulative blood volume discarded within a 6-hour period may reach 21–35 mL. Furthermore, when the additional blood volume required for laboratory analyses is factored in, the total daily blood loss is substantially increased.

For pediatric patients, particularly those with low body weight, frequent and substantial blood loss may increase the risk of iatrogenic anemia (11, 12). Notably, current research on drug concentration obtained from blood sampling through CVC has primarily focused on cyclosporine, tacrolimus, and similar drugs (13–15), with no studies specifically addressing the optimal discard volume for plasma Busulfan concentration. Given the unique characteristics of Busulfan TDM (requiring multiple blood samplings within a short time period) and the distinctive physiological features of pediatric patients, exploring the minimum discard volume necessary to ensure the accuracy of Busulfan concentration holds significant clinical implications.

This study aims to evaluate the impact of different discard volumes on the accuracy of plasma Busulfan concentration by comparing blood samples collected from double-lumen CVC with discard volumes of 1 mL, 2 mL, and 3 mL to those collected with the current clinically used discard volume of 4 mL. The ultimate goal is to minimize unnecessary blood loss associated with repeated blood sampling while ensuring the accuracy and reliability of plasma Busulfan concentration measurements. The findings will provide evidence for optimizing Busulfan TDM in pediatric HSCT patients, contributing to reducing unnecessary blood loss and lowering risk of iatrogenic anemia, while ensuring the accuracy and reliability of plasma Busulfan concentration. This will, in turn, advance the development and improvement of CVC care practices.

## 2 Materials and methods

This study was approved by the Institutional Ethics Committee (Approval No.: 202403302). Written informed consent was obtained from the parents or legal guardians of all participants following a thorough explanation of the study's procedures and potential risks. The study design minimized additional blood collection by utilizing samples that would otherwise have been discarded during routine clinical care. All personal identifiers were removed to ensure patient confidentiality. Participants and their guardians were informed of their right to withdraw from the study at any time without affecting the quality or continuity of their standard medical care. The entire consent process adhered to the ethical principles outlined in the Declaration of Helsinki. This study was not designed as a clinical trial and therefore does not have a clinical trial registration number.

### 2.1 Study design and participants

This was a prospective cohort study. Pediatric patients who underwent HSCT and required Busulfan chemotherapy from July and October 2024 were enrolled as study subjects. The inclusion criteria were as follows: (1) patients undergoing HSCT; (2) patients requiring blood sampling for plasma Busulfan concentration; (3) patients receiving Busulfan infusion through a double-lumen 5F non-tunneled CVC; (4) patients with patent double-lumen CVCs; and (5) patients whose guardians provided written informed consent. The exclusion criteria were as follows: (1) patients with single-lumen CVC; and (2) patients requiring peripheral venous blood sampling for plasma Busulfan concentration. Patients were withdrawn from the study if unplanned CVC removal or occlusion occurred prior to blood sampling for plasma Busulfan concentration measurement. This study adhered to the STROBE guidelines for reporting observational studies (16).

### 2.2 Data collection and sampling procedures

Relevant data of the patients were collected within 24 h of admission. Baseline demographic information, including age, gender, and disease-related parameters, was extracted from medical records. Blood samples were collected by nurses who received standardized training 0, 1, 2, 2.5, 3, 4, and 6 h after the fourth dose of Busulfan infusion, yielding seven sampling time points per patient. The 4 mL discard volume typically required in clinical practice was selected for plasma Busulfan concentration measurement, without increasing the total blood sampling volume from pediatric patients. The specific sampling procedure was as follows: First, both infusion pathways were clamped. Busulfan was administered as an intravenous infusion for 2 h via one lumen of the double-lumen CVC. Blood samples were collected from the non-infusion lumen (i.e., the lumen not used for Busulfan administration) to avoid direct drug contamination. This lumen was flushed with 10 mL of 0.9% sodium chloride solution before sampling^6^. Subsequently, an initial 1 mL blood sample was collected and discarded. Then, four consecutive 1 mL blood samples were collected for analysis without interruption. This design minimized blood loss by analyzing blood that would otherwise be discarded. Under standard practice, 4 mL is discarded at each sampling time point (28 mL total across 7 time points). In our study, only 1 mL was discarded per time point, and the remaining blood that would have been wasted was analyzed as the four study groups. Each patient contributed 35 mL total the same volume required for routine therapeutic drug monitoring. No additional blood was drawn for research purposes. Three were transferred to different tubes, serving as the samples in “Observation Group 1”, “Observation Group 2”, and “Observation Group 3”, respectively. The fourth sample was transferred to a tube labeled “Control Group” for routine plasma Busulfan concentration. This sequential sampling method resulted in different total discard volumes of 1 mL, 2 mL, 3 mL, and 4 mL (the standard clinical practice), respectively (Figure 1). Plasma Busulfan concentrations and exposure values were compared between the observation groups and the control group.

**Figure 1:**
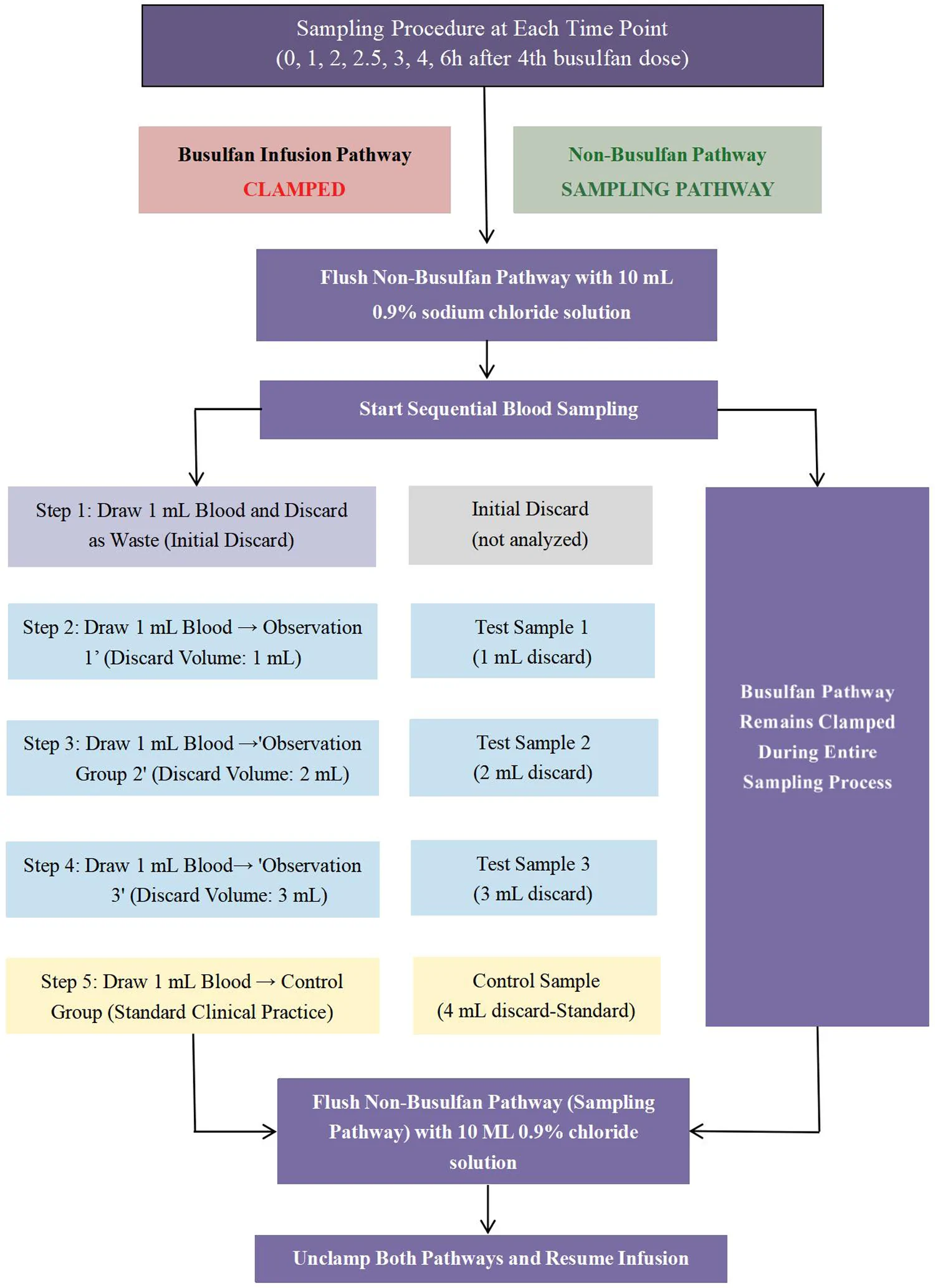
Sequential blood sampling procedure using dual-lumen CVC to compare different discard volumes. This schematic illustrates the dual-lumen CVC sampling technique used in this study. At each sampling time point (0, 1, 2, 2.5, 3, 4, and 6 h after the 4th Busulfan dose), the Busulfan infusion pathway was clamped while the non-Busulfan pathway was flushed and used for sequential blood collection. After discarding the initial 1 mL draw (Step 1), three consecutive 1 mL blood samples were collected representing Observation Groups with cumulative discard volumes of 1 mL (Step 2), 2 mL (Step 3), and 3 mL (Step 4). A final control sample was drawn after the standard 4 mL discard volume (Step 5), following conventional clinical practice. This within-subject design enabled direct comparison of Busulfan plasma concentrations across different discard volumes while the Busulfan pathway remained clamped throughout sampling to prevent drug contamination. After completion, the sampling pathway was flushed and both lumens were unclamped to resume infusion.

### 2.3 Analytical method for Busulfan

Blood samples for Busulfan analysis were collected in ethylenediaminetetraacetic acid (EDTA) tubes and centrifuged at 4 ℃ and 4,000 rpm within 2 h to obtain plasma. Fifty microliters of plasma were transferred to a 1.5 mL Eppendorf tube, 20 μL of Busulfan-D8 internal standard and 300 μL of methanol were added, and the mixture was vortexed for 5 min. After centrifugation at 15,000 rpm for 5 min, 200 μL of the supernatant was transferred to a 96-well plate and 2 μL was injected for analysis. Concentrations were quantified by high-performance liquid chromatography-tandem mass spectrometry (HPLC-MS/MS) on a Sciex 4500MD system. Chromatographic separation was performed on a Waters XBridge Premier BEH C18 column (2.5 µm, 2.1 × 100 mm) at 40 ℃ with a flow rate of 0.4 mL/min using gradient elution. The lower limit of quantification (LLOQ) was 50 ng/mL, and both inter- and intra-assay coefficients of variation (CV) were <10%. This method has undergone complete methodological validation and has been shown to accurately quantify Busulfan concentrations in human plasma. All samples from the same patient were analysed within the same batch to minimise variability. Pharmacokinetic parameters, including the AUC, were calculated by non-compartmental analysis.

### 2.4 Sample size

The non-inferiority margin (Δ) for AUC was prospectively prespecified as 6% of the pilot/reference AUC of 845.50 μmol/L·min, corresponding to 50.73 μmol/L·min. This prespecified margin was determined before analysis of the present study and was therefore independent of the observed AUC values obtained in the current study. For the paired contrast defined as reduced discard-volume AUC minus 4 mL reference AUC, the corresponding non-inferiority boundary was −Δ (−50.73 μmol/L·min). The 6% margin was selected as a conservative analytical tolerance for Busulfan exposure in the context of therapeutic drug monitoring, where maintaining reliable AUC estimation is important for dose individualization. The margin was intended to permit a small analytical difference associated with reducing the discard volume while avoiding a clinically meaningful change in estimated Busulfan exposure. This study therefore used the 6% margin prospectively to evaluate whether reduced discard volumes could provide AUC measurements sufficiently comparable to the standard 4 mL discard condition. In the pilot data used for sample-size planning, the mean paired differences [± standard deviation (SD)] between the 4 mL reference condition and the 1 mL, 2 mL, and 3 mL discard-volume conditions were 220.09 ± 125.48 μmol/L·min, 146.01 ± 151.10 μmol/L·min, and 137.91 ± 154.32 μmol/L·min, respectively. These pilot estimates of the paired differences and their corresponding SDs, together with the prespecified non-inferiority margin (Δ = 50.73 μmol/L·min), were entered into the “Non-Inferiority Tests for Paired Means” procedure in PASS 2021. A 6% allowance for missing data was applied. The minimum required sample size was 13 patients, with each patient contributing paired observations under all four discard-volume conditions. The PASS 2021 calculation was performed using a one-sided significance level (*α* = 0.025) with a target power of 80%. The sample size calculation followed a one-sided non-inferiority testing framework. In the final analysis, non-inferiority was evaluated using the lower bound of the two-sided 95% confidence interval for the paired mean difference. This approach is statistically equivalent to a one-sided *α* = 0.025 test and provides a conservative confidence-interval-based assessment of non-inferiority.

### 2.5 Statistical analysis

All statistical analyses were performed using R software (version 4.2.2). Continuous variables were presented as mean (SD) or median (interquartile range), while categorical variables were summarized as frequencies (percentages). Considering the repeated-measures and within-subject design of the study, comparisons between reduced discard volumes (1 mL, 2 mL, and 3 mL) and the reference 4 mL discard volume were performed using paired analyses within the same patient and sampling time point. Paired comparisons were performed to account for the within-patient nature of the repeated measurements. For AUC analysis, values were calculated at the patient level (*n* = 13 per discard-volume condition), and paired non-inferiority analyses were performed using the predefined non-inferiority margin. AUC values were calculated for each patient under each discard-volume condition (*n* = 13 per condition). Paired comparisons between each reduced discard volume (1 mL, 2 mL, and 3 mL) and the 4 mL reference condition were performed using paired non-inferiority analyses. The paired difference was defined as the AUC for the reduced discard volume minus the AUC for the 4 mL reference condition. The non-inferiority margin (*Δ*) was prespecified as 50.73 μmol/L·min. Accordingly, the null hypothesis was H₀: μ_difference ≤ −*Δ*, and the alternative hypothesis was H₁: μ_difference > −Δ. Non-inferiority was concluded when the lower bound of the two-sided 95% confidence interval for the paired mean difference was greater than −Δ (−50.73 μmol/L·min). ICC was calculated to assess measurement agreement between each reduced discard-volume condition and the 4 mL reference condition. Bland–Altman 95% limits of agreement were calculated from the observed paired differences as the mean difference ± 1.96 × SD of the paired differences. The prespecified non-inferiority boundary of −50.73 μmol/L·min was derived separately from the predefined non-inferiority margin and was not used as a Bland–Altman limit of agreement.

## 3 Results

### 3.1 Baseline characteristics

A total of 13 pediatric patients undergoing hematopoietic stem cell transplantation (HSCT) were enrolled in the study. The mean age was 6.22 ± 3.26 years, ranging from 1.8 to 12.3 years. The cohort included 8 females (61.5%) and 5 males (38.5%). Most patients (*n* = 10, 76.9%) were diagnosed with *β*-thalassemia major, while the remaining 3 patients (23.1%) had other hematological disorders. The average body weight was 20.59 ± 9.03 kg. All patients had double-lumen 5F non-tunneled central venous catheters (CVCs), with a mean catheter duration of 8.23 ± 1.92 days. No catheter-related complications or sample losses occurred during the study. Baseline demographic and clinical data are summarized in Table 1.

**Table 1:** Baseline characteristics of the study population.

Item	n (%)	Missing
Age [years, mean (SD)]	6.22 (3.26)	0
Gender (%)		0
Female	8 (61.5)	–
Male	5 (38.5)	–
Diagnosis (%)		0
AA	1 (7.7)	–
ALL	1 (7.7)	–
PKD	1 (7.7)	–
TM	10 (76.9)	–
Weight [kg, mean (SD)]	20.59 (9.03)	0
Duration of CVC insertion [days, mean (SD)]	8.23 (1.92)	0
Busulfan exposure (ng/mL)		
T0 [mean (SD)]	299.67 (90.36)	0
T1 [mean (SD)]	540.44 (115.08)	0
T2 [mean (SD)]	776.60 (163.78)	0
T3 [mean (SD)]	848.87 (164.96)	0
T4 [mean (SD)]	753.90 (160.53)	0
T5 [mean (SD)]	525.17 (119.45)	0
T6 [mean (SD)]	278.44 (87.16)	0
Total [mean (SD)]	3,328.78 (677.97)	0

AA, aplastic anemia; ALL, acute lymphoblastic leukemia; PKD, pyruvate kinase deficiency; TM, *β*-thalassemia major; CVC, central venous catheter; AUC, area under the concentration-time curve. Total represents the summed Busulfan concentration measurements across all seven sampling time points (T0–T6).

### 3.2 Comparison of plasma Busulfan concentrations across discard volume groups

A total of 364 plasma Busulfan concentration measurements were analyzed from 13 patients. Because each patient underwent sequential sampling at seven time points, comparisons between reduced discard volumes and the 4 mL reference were performed using paired analyses. At each sampling time point, 13 patients contributed paired observations under each discard-volume condition. Therefore, although 91 repeated measurements were available for each discard-volume condition across the seven sampling time points, the paired comparisons were based on the 13 patients rather than treating the repeated measurements as independent observations. Paired differences between 1 mL, 2 mL, and 3 mL discard volumes compared with the 4 mL reference remained small and the corresponding 95% confidence intervals demonstrated consistent agreement (all *p* > 0.05; Table 2). The paired non-inferiority analysis of AUC is presented in Table 3. AUC was analyzed at the patient level (*n* = 13 per discard-volume condition), with each reduced discard volume (1 mL, 2 mL, and 3 mL) compared with the 4 mL reference condition. The paired difference was defined as reduced discard-volume AUC minus 4 mL reference AUC. The paired mean differences and corresponding 95% confidence intervals were evaluated against the prespecified non-inferiority boundary of −50.73 μmol/L·min. Because the paired difference was defined as reduced discard-volume AUC minus 4 mL reference AUC, non-inferiority was concluded when the lower bound of the two-sided 95% confidence interval exceeded −50.73 μmol/L·min. The lower bounds were −18.87, −19.60, and −33.19 μmol/L·min for the 1 mL, 2 mL, and 3 mL comparisons, respectively, all exceeding −50.73 μmol/L·min. Thus, all three reduced discard-volume conditions were non-inferior to the standard 4 mL discard volume. These findings indicate that reducing the discard volume from the standard 4 mL to as low as 1 mL does not affect measured Busulfan concentrations or alter the overall pharmacokinetic exposure profile.

**Table 2:** Comparison of plasma Busulfan plasma concentrations among different discard blood volume groups.

Time points	Discharged blood volume (*n* = 364)				*P* value	Missing
	1 mL (*n* = 13 patients)	2 mL (*n* = 13 patients)	3 mL (*n* = 13 patients)	4 mL (*n* = 13 patients)		
T0	299.95 (98.26)	297.49 (96.13)	295.00 (93.84)	299.67 (90.36)	0.987	0
T1	541.62 (130.80)	542.83 (114.03)	533.74 (111.54)	540.44 (115.08)	0.956	0
T2	780.66 (169.60)	774.23 (161.21)	764.48 (159.13)	776.60 (163.78)	0.934	0
T3	847.79 (170.87)	846.88 (170.93)	834.93 (155.42)	848.87 (164.96)	0.945	0
T4	760.21 (162.64)	753.80 (161.22)	733.12 (158.32)	753.90 (160.53)	0.812	0
T5	519.47 (125.00)	516.66 (120.95)	510.20 (120.17)	525.17 (119.45)	0.891	0
T6	274.73 (84.02)	274.33 (83.87)	270.12 (84.36)	278.44 (87.16)	0.923	0

Data are presented as mean (SD). Each of the 13 patients contributed measurements at seven sampling time points (T0–T6), resulting in 91 repeated measurements per discard-volume condition overall. However, the patient remained the unit of paired analysis, and statistical comparisons were performed using within-patient paired observations.

**Table 3:** Paired Non-inferiority analysis of AUC for reduced discard volumes compared with the 4 mL reference condition.

Comparison	N	Mean AUC, reduced discard volume	Mean AUC, 4 mL reference	Paired mean difference	95% CI	Non-inferiority boundary (−*Δ*)	Conclusion
1 mL vs. 4 mL	13	867.36	857.38	9.98	−18.87 to 38.83	−50.73	Non-inferior
2 mL vs. 4 mL	13	862.41	857.38	5.03	−19.60 to 29.66	−50.73	Non-inferior
3 mL vs. 4 mL	13	849.67	857.38	−7.71	−33.19 to 17.76	−50.73	Non-inferior

Paired mean difference = reduced discard-volume AUC−4 mL reference AUC. The prespecified non-inferiority margin was *Δ* = 50.73 μmol/L·min, corresponding to 6% of the pilot/reference AUC of 845.50 μmol/L·min. Because the contrast was defined as reduced discard-volume AUC minus 4 mL reference AUC, the corresponding non-inferiority boundary was −*Δ* = −50.73 μmol/L·min. Non-inferiority was concluded when the lower bound of the two-sided 95% confidence interval exceeded −50.73 μmol/L·min.

### 3.3 Agreement analysis

#### 3.3.1 ICC

To assess the reliability of measurements across discard volume groups, ICCs were calculated using the 4 mL discard volume (standard clinical practice) as the reference. The ICCs for plasma Busulfan concentrations at each of the seven time points ranged from 0.983 to 0.998 across all comparison groups (Observation Group 1–3 vs. Control Group), indicating excellent agreement (all *p* < 0.001). Likewise, the ICC values for AUC comparisons between the observation groups and the control group were all above 0.98, further confirming the robustness and consistency of results irrespective of discard volume **(**Figure 2). A representative patient's concentration-time profile (Supplementary Figure S1) visually confirms these findings, with all four discard volume curves overlapping throughout the sampling period.

**Figure 2:**
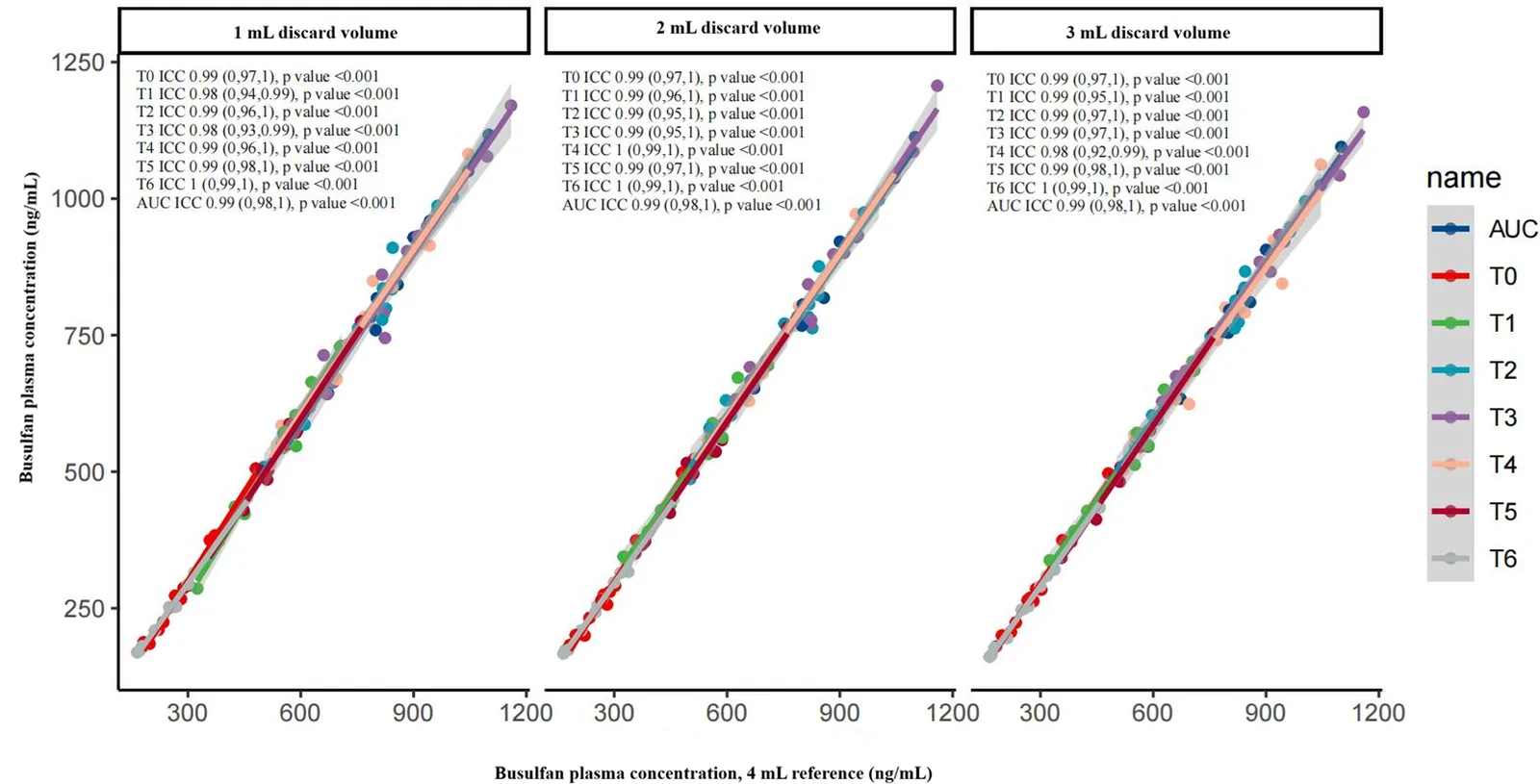
Correlation of Busulfan plasma concentrations and AUC values among different discard volumes. Correlation between test volumes (1 mL, 2 mL, 3 mL) and the reference volume (4 mL) for Busulfan concentrations at sampling time points (T0–T6) and AUC. ICC values with *p*-values are shown for each correlation. AUC, area under the concentration-time curve.

#### 3.3.2 Bland-Altman analysis

Bland–Altman plots were constructed to evaluate the agreement between reduced discard volume groups (1 mL, 2 mL, and 3 mL) and the standard 4 mL control. The plots revealed that the differences in measured Busulfan concentrations and AUC values were symmetrically distributed around the mean and remained within the calculated 95% limits of agreement. No systematic bias or trend was observed across time points or between discard volume groups. The agreement between 1 mL and 4 mL discard volumes was particularly strong, with the narrowest spread of differences observed in this comparison (Figure 3, Supplementary Figures S2, S3). These results indicate that a discard volume of as low as 1 mL is sufficient to obtain accurate plasma Busulfan concentrations and drug exposure profiles, thereby supporting the potential for blood conservation in pediatric clinical practice without compromising pharmacokinetic monitoring quality.

**Figure 3:**
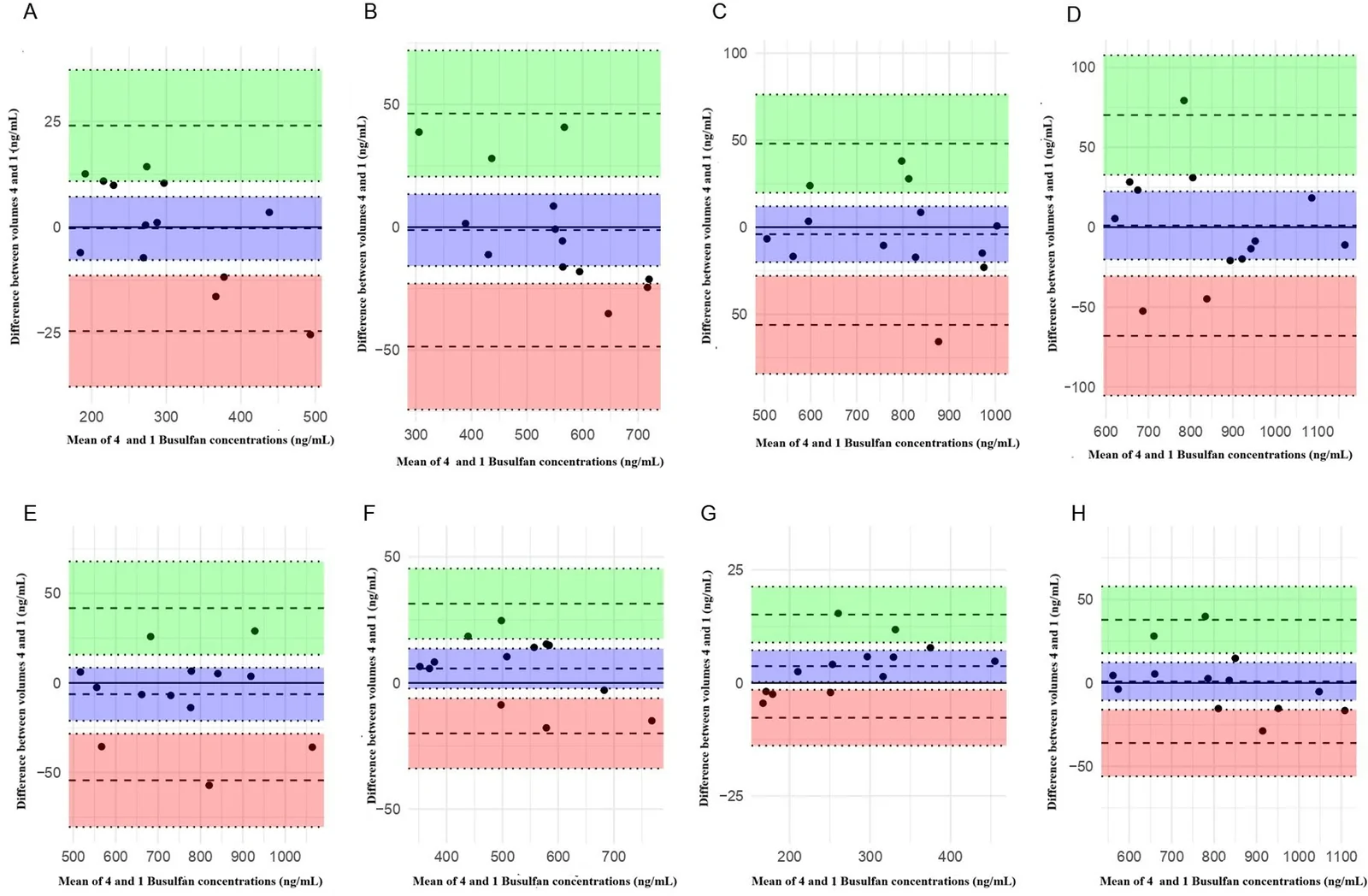
Bland-Altman plots for Busulfan concentrations at different time points and AUC values between 1 mL and 4 mL discard volume. Bland-Altman plots comparing 1 mL versus 4 mL discard volumes. **(A-G)**: agreement for plasma Busulfan concentrations at time points T0–T6, and **(H)**: agreement for AUC values.

## 4 Discussion

Accurate TDM of Busulfan is critical in pediatric HSCT to ensure optimal dosing, minimize toxicity, and improve transplant outcomes (17). However, in young patients with limited blood volume, repeated sampling can contribute to cumulative blood loss, particularly when large discard volumes are used prior to obtaining samples from central venous catheters. This study is the first to systematically evaluate the impact of different discard volumes prior to blood sampling via double-lumen CVCs on the accuracy of plasma Busulfan concentration measurements in pediatric patients undergoing HSCT. The results demonstrated no statistically significant differences in plasma Busulfan concentrations across discard volumes of 1 mL, 2 mL, 3 mL, and 4 mL at all time points (T0-T6) or in overall drug exposure AUC (*p* > 0.05). Furthermore, ICC analysis and Bland-Altman analysis confirmed the consistency between plasma Busulfan concentration obtained with a 1 mL discard volume and the standard 4 mL discard volume. The ICC values were ≥0.98 (*p* < 0.001), and measurement differences were uniformly distributed within the limits of agreement, with no systematic bias. These findings indicate that a 1 mL discard volume can provide comparable accuracy to the standard 4 mL discard volume for plasma Busulfan concentration.

Recent years have witnessed an increase in studies on discard volume for blood sampling through CVC (18). In a study involving 64 adult patients, no significant differences were observed in serum sodium, potassium, calcium, creatinine, direct bilirubin, and total bilirubin levels when comparing 3 mL and 5 mL discard volumes using triple-lumen catheters (8). Another study on discard volumes for triple-lumen CVCs demonstrated that a 2 mL discard volume was sufficient to ensure accurate results for complete blood count, coagulation tests, and chemistry panels (19). Meanwhile, the study selected the proximal lumen for blood sampling while vasoactive drugs and parenteral nutrition were delivered through the middle and distal lumens. This method prevented contamination of blood samples, which could affect the accuracy of blood test results (19). In comparison with previous studies, which focused mainly on adult populations using triple-lumen central venous catheters or central venous access devices and evaluated conventional laboratory parameters, our study focused on pediatric patients undergoing Busulfan TDM. Previous investigations demonstrated the reliability of 2 mL and 3 mL discard volumes, respectively; however, our study further reduced the discard volume to 1 mL, supporting the feasibility of minimizing blood loss during therapeutic drug monitoring in pediatric clinical practice (18, 20).

Furthermore, the latest “Infusion Therapy Standards of Practice” guidelines recommend that blood discard volumes be individualized based on catheter type and patient characteristics to minimize unnecessary blood loss (21). Similarly, Wyant and Crickman (11) proposed that blood sampling should minimize unnecessary blood wast (11). These recommendations align with the focus of our study. Our study adopted non-tunneled double-lumen CVC, which demonstrated that a 1 mL discard volume did not affect plasma Busulfan concentration. In contrast, previous investigations in adult patient populations reported that larger discard volumes may be required depending on catheter type, with non-tunneled and tunneled central venous catheters requiring discard volumes of approximately 6 mL and 9 mL, respectively (11). These differences may be related to variations in patient characteristics, catheter design, catheter volume, sampling procedures, and study methodologies. However, subsequent studies have demonstrated that lower discard volumes can provide reliable laboratory results while minimizing unnecessary blood loss. Collectively, these findings highlight the importance of optimizing discard volumes according to clinical context and analytical requirements, particularly in vulnerable populations where repeated blood sampling may contribute to clinically significant blood loss.

Despite ongoing advancements in CVC blood sampling, research on discard volumes for TDM remains limited. Jennifer Chen's study demonstrated that the push-pull method constitutes a reliable and safe approach for blood sampling of plasma Gentamicin concentrations (22). However, further investigation is needed to determine whether this method induces hemolysis that may affect the accuracy of plasma drug concentrations. A study on CVC in adults found that a 5-mL discard volume provided accurate plasma Cyclosporine concentrations (23). However, this study was limited to adults and did not include pediatric patients. Moreover, cyclosporine as a highly lipophilic drug that tends to adhere to catheter surfaces may have different requirements compared to Busulfan, suggesting that optimal discard volumes might be drug-specific rather than universally applicable. Furthermore, to date, no studies have specifically investigated the optimal discard volume for plasma Busulfan concentrations, making our investigation the first to explore the impact of varying discard volumes on plasma Busulfan concentration when sampling through CVC. Our study aims to determine the minimum discard volume that ensures measurement accuracy while minimizing unnecessary blood loss. Importantly, our study focuses on pediatric patients, a population particularly vulnerable to iatrogenic blood loss from diagnostic phlebotomy.

Busulfan TDM represents a high blood loss scenario due to multiple required draws (7 samples = 28 mL discarded with standard practice). Our finding that 1 mL discard is sufficient could reduce this to 7 mL—a 75% reduction. While this study focused on Busulfan, we validated a fundamental pre-analytical principle: whether reduced discard volume adequately clears catheter dead space to prevent sample contamination. Although the present study focused specifically on Busulfan therapeutic drug monitoring, the underlying pre-analytical question may be relevant to other blood tests obtained through CVCs. However, the applicability of reduced discard volumes to other analytes, catheter types, and clinical settings requires independent validation. Importantly, this study was designed as an analytical validation study rather than a clinical outcome trial. Because all patients underwent the same standard sampling protocol and total blood volume collection remained unchanged, direct comparison of hemoglobin changes or anemia outcomes between discard volumes was not feasible. Therefore, hemoglobin and hematocrit were not used as outcome measures in this study because the objective was to evaluate analytical accuracy of Busulfan therapeutic drug monitoring rather than clinical hematological outcomes. Future prospective studies implementing reduced discard-volume protocols should evaluate changes in hematological parameters and transfusion requirements.

Despite these promising results, our study is subject to several limitations. Specifically, it was restricted to pediatric patients, and analyses focused solely on plasma Busulfan concentration. As such, the applicability of these findings to adult patients and other laboratory tests requires further investigation. Additionally, the results may not be generalizable across different catheter types. While this study was conducted at a single center with a relatively small and clinically homogeneous cohort primarily pediatric patients with *β*-thalassemia major, it provides a strong proof-of-concept for the safe reduction of discard volume during Busulfan therapeutic drug monitoring. The consistent results across all time points, supported by robust statistical agreement, highlight the feasibility of implementing blood-conserving strategies in similar clinical settings. These findings lay the groundwork for future multicenter studies involving broader pediatric populations, a wider range of catheter types, and additional therapeutic agents. Such investigations will be essential to further validate and extend these observations, ultimately supporting the development of optimized, evidence-based guidelines for blood sampling in pediatric pharmacokinetics and therapeutic monitoring. Additionally, while our study validates reduced discard volumes using standard laboratory assays, future research should explore alternative sampling methods such as dried blood spots or volumetric microsampling devices to further minimize blood volume requirements in pediatric populations.

## 5 Conclusion

This study systematically investigated the influence of varying discard volumes on the accuracy of plasma Busulfan concentration measurements obtained via double-lumen central venous catheters (CVCs) in pediatric patients undergoing hematopoietic stem cell transplantation. The findings demonstrate that a reduced discard volume of 1 mL yields plasma Busulfan concentrations and AUC values that are statistically and clinically comparable to those obtained using the standard 4 mL discard volume. Across all seven sampling time points, no significant differences were observed in Busulfan concentrations among the different discard-volume groups. High intraclass correlation coefficients (ICCs) and consistent Bland–Altman agreement further supported the reliability and agreement of measurements obtained with reduced discard volumes, with no indication of systematic measurement bias. These results suggest that reducing the discard volume to 1 mL does not compromise the accuracy of Busulfan therapeutic drug monitoring in this pediatric cohort. This approach may help reduce unnecessary blood loss associated with repeated blood sampling, particularly in pediatric patients requiring frequent therapeutic drug monitoring; however, the direct effect on iatrogenic anemia or transfusion requirements was not evaluated in this study. While Busulfan therapeutic drug monitoring served as the validation model because of its frequent sampling requirements, the applicability of this discard-volume approach to other laboratory analytes, catheter types, patient populations, and clinical settings remains to be established and requires further validation.

## Data Availability

The original contributions presented in the study are included in the article/Supplementary Material, further inquiries can be directed to the corresponding author/s.

## References

[1] Soh KVQY Hwang WYK. Optimizing blood stem cell transplants through cellular engineering. Blood Cell Ther. (2022) 5:1–15. 10.31547/bct-2021-008 36714264 PMC9847292

[2] Du X Huang C Xue L Jiao Z Zhu M Li J. The correlation between Busulfan exposure and clinical outcomes in Chinese pediatric patients: a population pharmacokinetic study. Front Pharmacol. (2022) 13:905879. 10.3389/fphar.2022.905879 35784763 PMC9243314

[3] Bartelink IH Lalmohamed A Van Reij EML Dvorak CC Savic RM Zwaveling J. Association of Busulfan exposure with survival and toxicity after haemopoietic cell transplantation in children and young adults: a multicentre, retrospective cohort analysis. Lancet Haematol. (2016) 3:e526–36. 10.1016/S2352-3026(16)30114-4 27746112 PMC5159247

[4] Alomari N Kurdi A Alghamdi M Alhussaini W Ezzeldeen G Alharbi E. Therapeutic drug monitoring of Busulfan in pediatric patients: a 5-year observational study. BMC Pharmacol Toxicol. (2025) 26:175. 10.1186/s40360-025-01015-w 41152998 PMC12570591

[5] These highlights do not include all the information needed to use BUSULFEX® safely and effectively. See full prescribing information for BUSULFEX. BUSULFEX (busulfan) Injection, for intravenous use Initial U.S. Approval (1999). Available online at: https://dailymed.nlm.nih.gov/dailymed/fda/fdaDrugXsl.cfm?setid=5cb9d285-1803-4a99-946a-d0b239b32df6 (Accessed August 14, 2026).

[6] Rondina MT Markewitz B Kling SJ Nohavec R Rodgers GM. The accuracy of activated partial thromboplastin times when drawn through a peripherally inserted central catheter. Am J Hematol. (2007) 82:738–9. 10.1002/ajh.20900 17492643

[7] Gorski LA Hadaway L Hagle ME Broadhurst D Clare S Kleidon T. Infusion therapy standards of practice, 8th edition. J Infus Nurs. (2021) 44:S1–S224. 10.1097/NAN.0000000000000396 33394637

[8] Lalthanthuami HT Kumari MJ Venkateswaran R Lakshmi PR Ramamoorthy L. Performance of 3 mL versus 5 mL discarded volume for blood sampling from central venous access device. J Lab Physicians. (2021) 13:112–7. 10.1055/s-0041-1726669 34483554 PMC8409120

[9] Spina R Mussa B Tollapi L Conti F Cortesi E Verna R. Adoption and application in Italy of the principal guidelines and international recommendations on venous access. Minerva Med. (2018) 109:153–202. 10.23736/S0026-4806.18.05552-0 29849020

[10] Li C Wu X Feng X He Y Liu H Pei F. A novel conditioning regimen improves outcomes in *β*-thalassemia major patients using unrelated donor peripheral blood stem cell transplantation. Blood. (2012) 120:3875–81. 10.1182/blood-2012-03-417998 22968457

[11] Wyant S Crickman R. Determining the minimum discard volume for central venous catheter blood draws. Clin J Oncol Nurs. (2012) 16:454–8. 10.1188/12.CJON.454-458 23022928

[12] Bateman ST Lacroix J Boven K Forbes P Barton R Thomas NJ. Anemia, blood loss, and blood transfusions in north American children in the intensive care unit. Am J Respir Crit Care Med. (2008) 178:26–33. 10.1164/rccm.200711-1637OC 18420962

[13] Ting LSL Villeneuve E Ensom MHH. Beyond cyclosporine: a systematic review of limited sampling strategies for other immunosuppressants. Ther Drug Monit. (2006) 28:419–30. 10.1097/01.ftd.0000211810.19935.44 16778729

[14] Grouzmann E Buclin T Biollaz J. Misleading tacrolimus concentration value in blood taken from a catheter used for tacrolimus administration. Am J Health Syst Pharm. (2008) 65:226–8. 10.2146/ajhp070054 18216007

[15] Marschner MN Chandran MM Colyer LG. Artificially elevated tacrolimus concentrations obtained from a venous catheter previously used for tacrolimus administration in a pediatric patient. J Pharm Pract. (2023) 36:1264–7. 10.1177/08971900221107841 35689341

[16] Von Elm E Altman DG Egger M Pocock SJ Gøtzsche PC Vandenbroucke JP, STROBE Initiative. The strengthening the reporting of observational studies in epidemiology (STROBE) statement: guidelines for reporting observational studies. J Clin Epidemiol. (2008) 61:344–9. 10.1016/j.jclinepi.2007.11.008 18313558

[17] Ben Hassine K Gloor Y Dupanloup I Uppugunduri CRS Mlakar V Gonzales F. Refining Busulfan exposure enhances pediatric ALL HSCT outcomes: insights from the international FORUM study. Blood Adv. (2026) 10:3719–30. 10.1182/bloodadvances.2025019142 41779961 PMC13216695

[18] Pushparaj A Ramamoorthy L S MA Murugesan R Lalthanthuami HT. Minimizing discard volume to 2 mL before blood sampling from a central venous catheter: a prospective observational study in India. Acute Crit Care. (2026) 41:174–82. 10.4266/acc.000792 41834640 PMC12989945

[19] Villalta-García P López-Herránz M Mazo-Pascual S Honrubia-Fernández T Jáñez-Escalada L Fernández-Pérez C. Reliability of blood test results in samples obtained using a 2-mL discard volume from the proximal lumen of a triple-lumen central venous catheter in the critically ill patient. Nurs Crit Care. (2017) 22:298–304. 10.1111/nicc.12220 26487571

[20] Castro-Olmo FJ Morales-Fernández P Alcaide-Martín MJ Fernández-Puntero B Quintana-Díaz M Aceña-Gil V. Is minimising waste volume for drawing blood samples in critically ill patients feasible? Enferm Intensiva (Engl Ed). (2023) 34:19–26. 10.1016/j.enfie.2022.06.002 36774248

[21] Nickel B Gorski L Kleidon T Kyes A DeVries M Keogh S. Infusion therapy standards of practice, 9th edition. J Infus Nurs. (2024) 47:S1–S285. 10.1097/NAN.0000000000000532 38211609

[22] Chen J Boodhan S Nanji M Chang A Sekharan S Lavoratore S. A reliable and safe method of collecting blood samples from implantable central venous catheters for determination of plasma gentamicin concentrations. Pharmacotherapy. (2011) 31:776–84. 10.1592/phco.31.8.776 21923604

[23] Garbin LM Simões BP Curcioli ACJV De Carvalho EC. Serum cyclosporine levels: the influence of the time interval between interrupting the infusion and obtaining the samples: a randomized clinical trial. Cancer Nurs. (2018) 41:E55–61. 10.1097/NCC.0000000000000544 29049039

