# Supplementary material for: Assessment of minimum discard volume for central venous catheter sampling: Validation via busulfan therapeutic drug monitoring in pediatric transplant patients
Source: Front Pediatr. 2026 Sep 14;14:1925821. doi: 10.3389/fped.2026.1925821 (PMC13617047; doi:10.3389/fped.2026.1925821)
Supplement: Supplementary file 1 [file Supplementaryfile1.docx]

**Supplementary material**

**Supplementary Figure 1.** **Plasma Busulfan concentration-time profiles for different discard blood volumes in a representative patient**

Note: *Data presented are from a single representative patient and are used to illustrate the consistency of Busulfan concentration profiles across different discard blood volumes. The overlapping curves demonstrate that discard volume does not significantly affect plasma Busulfan concentration measurements. This representative case reflects the overall trend observed across the entire patient cohort.*

**Supplementary Figure 2. Bland-Altman plots for Busulfan concentrations at different time points and AUC values between 2 mL and 4 mL discard volumes**

Note: *Bland-Altman plots comparing 2 mL versus 4 mL discard volumes. A-G: agreement for plasma Busulfan concentrations at time points T0-T6, and H: agreement for AUC values.*

**Supplementary Figure 3. Bland-Altman plots for Busulfan concentrations at different time points and AUC values between 3 mL and 4 mL discard volumes**

Note: *Bland-Altman plots comparing 3 mL versus 4 mL discard volumes. A-G: agreement for plasma Busulfan concentrations at time points T0-T6, and H: agreement for AUC values.*
